# Metformin represses the pathophysiology of AAA by suppressing the activation of PI3K/AKT/mTOR/autophagy pathway in ApoE^−/−^ mice

**DOI:** 10.1186/s13578-019-0332-9

**Published:** 2019-08-27

**Authors:** Zhu Wang, Jingjing Guo, Xinqiang Han, Ming Xue, Wenming Wang, Lei Mi, Yuguo Sheng, Chao Ma, Jian Wu, Xuejun Wu

**Affiliations:** 1 Department of Vascular Surgery, Shandong Provincial Hospital Affiliated to Shandong University, 324 Jing Wu Wei Qi Road, Jinan, 250021 Shandong China; 2 Department of Interventional Medicine and Vascular Surgery, Binzhou Medical University Hospital, Binzhou, 256603 Shandong China; 3 Department of Obstetrics and Gynecology, Binzhou Medical University Hospital, Binzhou, 256603 Shandong China; 4 Department of Interventional Radiology, Weihai Municipal Hospital, Weihai, 264200 Shandong China; 5 Department of General Surgery, Taian City Central Hospital, Taian, 271000 Shandong China

**Keywords:** Metformin, AAA, PI3K/AKT/mTOR pathway, Autophagy pathway, *ApoE*^−*/*−^ mice

## Abstract

**Background:**

The protective effect of metformin (MET) on abdominal aortic aneurysm (AAA) has been reported. However, the related mechanism is still poor understood. In this study, we deeply investigated the role of metformin in AAA pathophysiology.

**Methods:**

Angiotensin II (Ang-II) was used to construct the AAA model in *ApoE*^−*/*−^ mice. The related mechanism was explored using Western blot and quantitative real time PCR (qRT-PCR). We also observed the morphological changes in the abdominal aorta and the influence of metformin on biological behaviors of rat abdominal aortic VSMCs.

**Results:**

The PI3K/AKT/mTOR pathway was activated in aneurysmal wall tissues of AAA patients and rat model. Treatment with metformin inhibited the breakage and preserved the elastin structure of the aorta, the loss of collagen, and the apoptosis of aortic cells. In addition, metformin significantly suppressed the activation of the PI3K/AKT/mToR pathway and decreased the mRNA and protein levels of LC3B and Beclin1, which were induced by Ang-II. Moreover, PI3K inhibitors enhanced the effect of metformin while PI3K agonists largely reversed this effect. Interestingly, the cell proliferation, apoptosis, migration and autophagy of vascular smooth muscle cells (VSMCs) induced by Ang-II were also decreased following metformin treatment. PI3K inhibitors and agonists strengthened and weakened the effects of metformin in VSMCs, respectively.

**Conclusions:**

Metformin represses the pathophysiology of AAA by inhibiting the activation of PI3K/AKT/mTOR/autophagy pathway. This repression may be useful as a new therapeutic strategy for AAA.

## Introduction

AAA is a common chronic vascular degenerative disease which mainly occurs in elderly men over 60 years old [1]. In elderly populations, AAA becomes increasingly prevalent, and the maximum diameter of these tumors gradually increases, with a high risk of rupture [2]. Currently, due to the absence of effective medical treatment for AAA, high-risk surgical resection of AAA is generally performed to prevent rupture of the tumor [3]. Given the high morbidity, ineffective medical treatment and high surgical risk, it is of great significance to research the risk factors and the pathogenesis of AAA, so as to prevent the formation and rupture of AAA.

Metformin (MET) is a commonly used hypoglycemic drug in clinical practice [4]. In addition its effectiveness in producing a hypoglycemic effect and enhancing insulin sensitivity, it has anti-inflammatory, anti-oxidative stress effects and improves blood lipid and vascular endothelial function [5]. Recent reports have found that metformin could suppress AAA progression and prevalence in diabetic patients. Itoga et al. have demonstrated that the use of metformin in diabetic Veterans Affairs patients is closely related to the decrease of AAA enlargement [6]. Besides, Chaube et al. proved that metformin suppressed the complex I expression via up-regulating the levels of lactate and VEGF, thereby promoting the melanoma growth [7]. However, the mechanism of action of metformin in the progression of AAA still has not been reported.

Recently, with the in-depth study of vascular pathophysiology and molecular biology, it has been found that AAA is mainly due to the permanent expansion of the aortic wall, which is induced by the destruction of elastic proteins in the middle layer of the artery, the apoptosis of VSMCs and the deposition of compensatory collagen [8]. The inflammation and matrix degradation of the AAA vascular wall are key factors in the development of AAA [9, 10]. Therefore, in-depth exploration of immune and other mechanisms related to the pathogenesis of AAA is of great significance for the prevention and treatment of AAA. The PI3K-AKT-mTOR signaling pathway is an important tumor immune signaling pathway and regulates cell growth, proliferation, apoptosis and autophagy [11]. It has been demonstrated that many drugs can reduce AAA, which is induced by Ang-II through regulating the PI3K/Akt/mTOR and NF-κB signaling pathways [12]. However, the role of metformin in Ang-II-induced AAA is still unclear.

VSMCs are the major cellular components of the arterial wall. Upon stimulation of hemodynamics and the influence of various vascular injury factors, the structural integrity co-formed by the extracellular matrix (elastin and collagen) and VSMCs maintains the lumen diameter and blood flow continuity. Hyperplasia and apoptosis of VSMCs play an important role in pathophysiology of various cardiovascular diseases, including hypertension, aortic aneurysm, atherosclerosis and postoperative restenosis [13–16]. Both clinical and basic studies have proved that the loss of VSMCs is one of the important causes of aortic aneurysm formation [17].

In this study, we firstly identified the changes in PI3K/AKT/mTOR proteins expression in the aortic aneurysm tissue samples of AAA patients. Then Ang-II was used to construct the AAA model in ApoE^−/−^ mice. Based on this model, intervention treatment with metformin was used to investigate the effect of metformin on the occurrence and development of AAA and the role of PI3K/AKT/mTOR pathway in this process. The effect of metformin on cell proliferation, cell cycle, apoptosis, migration, invasion and autophagy of rat abdominal aortic VSMCs was also determined. In addition, PI3K inhibitors and agonists were used to further investigate the influence of metformin on the expression of genes and proteins of the PI3K/AKT/mTOR/autophagy pathway. The aim was to explore a potential therapeutic strategy to prevent and attenuate the progression of AAA.

## Materials and methods

### Clinical specimens

Ten patients (8 males and 2 females) with abdominal aortic aneurysm (AAA) that was diagnosed by computerized tomography (CT) and magnetic resonance imaging (MRI) were included in this study. The patients who underwent AAA resection and artificial blood vessel replacement in the vascular surgery department of Shandong Provincial Hospital (Shandong, China) were selected from January 2016 to December 2018. Their ages ranged from 49 to 72 years. The control group was consisted of 9 cases (7 males and 2 females) who provided redundant abdominal aortic tissues during kidney and liver transplantation in Shandong Provincial Hospital and Qilu Hospital of Shandong University (Shandong, China). The ages of patients in the control group ranged from 41 to 58 years old. The aneurysmal wall tissues of AAA and the aortic tissues from the control group were partly fixed with 4% paraformaldehyde and stored at − 80 °C. This study was approved by the Human Research Committee of Shandong Provincial Hospital affiliated to Shandong University. Procedures were carried out in accordance with the Declaration of Helsinki, and informed consent was obtained for experimentation with human subjects.

### Mouse model of Ang-II-induced aortic abdominal aneurysm

A total of 30 male C57BL/6 mice with ApoE^−/−^, 56 days old and pathogen free (SPF; certificate no. SCXK20140007) were purchased from the Animal Center of Jinan Pengyue Laboratory Animal Breeding Co., Ltd (Shandong, China) and housed in an animal experimental center. The mice were kept in standard conditions (22 ± 2 °C; 40–60% relative humidity) with a 12 h light/dark cycle. The ApoE-deficient mice at 10 weeks of age were weighed and randomly divided into three groups (N = 30). Mice in the AAA or sham group were infused with Ang-II (1.44 mg/kg/d) (Abcam, ab120183, UK) or normal saline, respectively, for 7 or 28 days by implanting micro-osmotic pumps. To understand the effect of metformin on the development of AAA, the ApoE-deficient mice at 10 weeks of age were infused with Ang-II (1.44 mg/kg/d) by implanting micro-osmotic pumps and feed water containing metformin (100 mg/kg/d) for 7 or 28 days [18]. The dosage of Ang-II solution was calculated according to 220 micro-liters/pump and allocated. A total of 200 microliters of normal saline or Ang-II solution were loaded into 1 ml syringe and then injected into the micro-osmotic pump. The assembled micro-osmotic pump was activated in a 37 °C water bath for 48 h after placement in a 50 mL centrifugal tube with PBS. The mice were weighed, anesthetized with sodium pentobarbital, fixed, shaved and disinfected with 70% ethanol. The microcosmic pump [normal saline (Sham group) or Ang-II solution (AAA model group)] was embedded in the scapular region, and the wound was sutured and disinfected again. The mice were fed in a cage alone after awakening. All procedures with C57BL/6 mice were approved by the Animal Care and Use Committee of Shandong Provincial Hospital and were conducted following the institutional guidelines. Ultrasound measurements of the abdominal aorta diameter were performed on day 28 to determine the effect of model building. After 28 days, mice were euthanized and AAA formation was evaluated.

### Cell line and culture

The rat thoracic aortic VSMCs were obtained from BeNa Culture Collection (BNCC340258, China) and cultured in Dulbecco’s modified Eagle’s medium (DMEM, Genview, USA) supplemented with 10% fetal bovine serum (FBS, Gibco), penicillin (100 U/mL) and streptomycin (100 U/mL). Cells were kept in a humidity incubator at 37 °C with 5% CO_2_ in atmosphere. When 80% confluence in culture wells was reached, the cells were used in treatment.

In the follow-up experiments, the VSMC were divided into five groups: (a) Control group; (b) Ang-II group (VSMC were treated by 1 µM Ang-II); (c) Ang-II and metformin group [VSMC were treated by Ang-II (1 µM) and metformin (10 mM)]; (d) Ang-II, metformin, and LY294002 (HY-10108, MedChemExpress, China) group [VSMC were treated by 1 µM Ang-II, 10 mM metformin together with LY294002 (10 µM)]; and (e) Ang-II, metformin, and 740 Y-P (HY-P0175, MedChemExpress, China) group (VSMC were treated by 1 µM Ang-II, 10 mM metformin together with 740 Y-P (25 mM)).

### Western blot analysis

The fold change of proteins in abdominal aortic vascular tissues and VSMCs were detected by Western blotting analysis. Protein lysates were extracted from abdominal aortic vascular tissues and VSMC cell lines using lysis buffer (Beyotime, China). This was followed by quantification and separation onto 10% sodium dodecyl sulfate-polyacrylamide gel electrophoresis (SDS-PAGE, Invitrogen, USA) and then transferred onto polyvinylidene fluoride (PVDF) membranes (Millipore, USA). The membranes were incubated with primary antibodies against AKT (CST, 1:1000), p-AKT (CST, 1:1000), mTOR (CST, 1:1000), p-mTOR (CST, 1:400), PI3K (CST, 1:1000), p-PI3K (CST, 1:3000), Beclin1 (CST, 1:1000), LC3B (CST, 1:500), Bcl-2 (CST, 1:1000), Bax (CST, 1:1000) or GAPDH (Abcam, 1:10,000). Horse radish peroxidase (HRP)-conjugated goat anti-rabbit secondary antibodies (BOSTER, 1:20,000) or HRP-conjugated goat anti-mouse secondary antibodies (BOSTER, 1:20,000) were used. Enhanced chemiluminescence technique (ECL, Amersham Biosciences, USA) was used for visualizing bands.

### Real-time PCR

Real-time PCR assay was used to investigate the expression of PI3K, AKT, mTOR, LC3B and Beclin1. Whole cellular RNA was extracted and reverse transcribed by Bestar qPCR RT Kit. The sequence of primers is shown in Table 1. To quantify gene amplification, real-time PCR analysis was performed using an Agilent Stratagene Mx3000P Sequence Detection System in the presence of Bestar^®^ SybrGreen (DBI Bestar, Germany). β-actin and GAPDH were used as internal controls for mouse tissues or VSMCs, respectively. The calculation method of the relative expression amount (defined as fold changes) was 2^−ΔΔCt^ [19].

**Table 1:** Primers for real-time RT-PCR analysis

ID	Sequence (5′–3′)	Product length (bp)
Mouse		
β-actin F	CATTGCTGACAGGATGCAGA	139
β-actin R	CTGCTGGAAGGTGGACAGTGA	
PI3K F	ACACCACGGTTTGGACTATGG	140
PI3K R	GGCTACAGTAGTGGGCTTGG	
AKT F	AGAAGAGACGATGGACTTCCG	111
AKT R	TCAAACTCGTTCATGGTCACAC	
mTOR F	CACCAGAATTGGCAGATTTGC	82
mTOR R	CTTGGACGCCATTTCCATGAC	
LC3B F	CGCTTGCAGCTCAATGCTAAC	93
LC3B R	CTCGTACACTTCGGAGATGGG	
Beclin1 F	ATGGAGGGGTCTAAGGCGTC	149
Beclin1 R	TGGGCTGTGGTAAGTAATGGA	
Rat		
GAPDH F	CCTCGTCTCATAGACAAGATGGT	169
GAPDH R	GGGTAGAGTCATACTGGAACATG	
PI3K F	TTAACCCCCTCACAGCAGAG	251
PI3K R	TTTCTGAACTGCAATGGCCC	
AKT F	GCTGCTCAAGAAGGACCCTA	218
AKT R	CTTGATCAGGCGGTGTGATG	
mTOR F	GTTTGAAGTGAAGCGAGCCT	231
mTOR R	CGCTGCGTGGTAAGAATCAG	
LC3B F	TACCAAGGCAAAAAGGGACG	282
LC3B R	CCCCTGACACTGCTCTTCTAT	
Beclin1 F	GACCGAGTGACCATTCAGGA	200
Beclin1 R	TGGTCTTCACAGGGTGCTAC	

### EdU staining

Staining with the thymidine analog 5-ethynyl-2′-deoxyuridine (EdU) was used to detect cell proliferation. The VSMCs were treated with 50 μM EdU (Ribobio, C10327, China) for 2 h. They were then detected with Apollo^®^ 643, and the nuclei were stained with DAPI (KeyGen, KGA215, China). Following this step, the cells were photographed using a fluorescence confocal microscopy (Olympus FV1000, Japan).

### Flow cytometric analysis

The influence of Ang-II, metformin and PI3K signaling pathway on VSMCs apoptosis and cell cycle distribution were detected after the corresponding treatment with VSMCs. Cell cycle distribution and apoptosis were detected by flow cytometry. For cell cycle distribution, cells were incubated with cell cycle staining kit (MultiSciences, CCS012, China) at room temperature for 30 min. For cell apoptosis, cells were incubated with Annexin V-FITC/PI apoptosis kit (MultiSciences, AP101, China) at room temperature for 15 min. The cell cycle distribution and apoptosis percentage were analyzed using the BD FACS Calibur flow cytometry (BD Biosciences).

### Immunofluorescence assay

Before the cell experiments began, VSMCs were identified via immunofluorescence using anti-α-SMA antibody. To research the effect of Ang-II, metformin and PI3K signaling pathway on VSMCs autophagy, the expression of LC3B in VSMCs was investigated by immunofluorescence. Briefly, VSMCs were fixed in 4% (w/v) paraformaldehyde for 30 min and permeabilized in Triton X-100 for 20 min. The cells were blocked with 10% non-fat milk and incubated with primary antibodies (anti-α-SMA, 1:100 dilution; anti-LC3B, 1:100 dilution) overnight at 4 °C, then stained with FITC-conjugated IgG (1:100 dilution) at room temperature for 2 h. Finally, the nucleus was stained with DAPI. Images were captured using a fluorescence microscope at 488 nm.

### Cell migration assay

To study the effect of Ang-II and metformin on cell migration in vitro, the wound healing assay and transwell chamber migration assay was used. For the wound healing assay, VSMCs were placed into 24-well plates and incubated for 24 h to allow for the formation of a monolayer on the bottom of the plate. Then, a straight line was scratched onto the monolayer using a 200-μL micropipette tip. After 48 h, the wound width was captured with a microscope (MOTIC) and analyzed by Image pro plus 6.0 (Media Cybernetics, USA). For the transwell assay, VSMCs (2 × 10^4^) were seeded into the upper chamber of a well. The cells were then allowed to migrate through the membranes for 48 h at 37 °C. Cells in the upper surface of the chamber were completely removed by cotton swabs, while cells on the lower surface of the membrane were fixed in 4% (w/v) paraformaldehyde for 20 min and stained with 0.5% (w/v) crystal violet for 5 min. Images were captured using a microscope and the number of cells on the membrane was counted.

### Transmission electron microscopy

To research the effect of Ang-II, metformin and PI3K signaling pathway on autophagy, transmission electron microscopy (TEM) was used. Briefly, VSMCs in each group were treated as previously described. Cells were collected, pelleted, pre-fixed in 2.5% glutaraldehyde (Sigma, USA) for more than 4 h at 4 °C, washed 3 times with PBS and postfixed with 1% osmium tetroxide (Sigma, USA) for 30 min at 4 °C. Samples were dehydrated at room temperature with a graded series of acetone (50%, 70%, 90% and 100% acetone), permeated with acetone/Epno812 (Shell Chemical, USA) at proportions of 1:1 for 30 min and finally permeated with 100% Epon812 for 2 h at room temperature. The samples were embedded in Epon812 and cured at 45 °C for 12 h and 60 °C for 36 h. Finally, ultra-thin (50–70 nm) sections were stained with lead citrate for 10 min, dyed with sodium acetate for 30 min, and observed with a Hitachi 600 (Hitachi, Japan) transmission electron microscope.

### HE, EVG, Masson and Sirius Red staining

HE, EVG, Masson and Sirius Red staining was performed according to the instructions. Briefly, paraffin slides (4 μm) were de-paraffinized in xylene I and II, each for 20 min, and dehydrated in 100% ethanol I and II for 10 min and followed by 95%, 90%, 80%, and 70% ethanol for 5 min, respectively. Then the slides were stained with hematoxylin and eosin (HE) solution (Servicebio, G1005, China), elastica van Gieson (EVG) (Servicebio, G1042, China), Masson (Servicebio, G1006, China), and Sirius Red (Servicebio, G1018, China) according to the respective instruction manuals. Images were captured using a microscope imaging system (Nikon, Japan).

### TUNEL staining

Briefly, paraffin slides (4 μm) were de-paraffinized in xylene and dehydrated in ethanol, respectively, before staining with TdT-mediated dUTP nick end labeling (TUNEL) (Roche, 11684817910, USA) according to the instruction manual. The nuclei were stained with DAPI. Images were captured using a fluorescence microscope (Olympus, Japan).

### Immunohistochemical staining

The expression of CD31, CD68, MMP-2, MMP-9, PI3K, AKT, mTOR, p-PI3K, p-AKT, p-mTOR, LC3B and Beclin1 in abdominal aortic tissues of mice were determined by immunohistochemical staining (IHC) according to the manufacturer’s guidelines. The abdominal aorta slides (4 μm) were incubated with antigen retrieval solution (Servicebio, G1203, China) and followed by 3% H_2_O_2_ for 25 min. Then the slides were blocked with 3% BSA and incubated with the primary antibody (CD31, CD68, PI3K, AKT mTOR, p-PI3K, p-AKT, p-mTOR, LC3B and Beclin1, respectively) overnight at 4 °C, and then HRP-conjugated secondary antibody at room temperature. The slides were incubated with diaminobenzidine (Dako, K5007, Denmark) and hematoxylin staining, respectively. Images were captured using a microscope imaging system (Nikon, Japan).

### Statistical analysis

All experiments were repeated at least three times. The data are presented as the mean ± standard deviation (SD). One-way analysis of variance (ANOVA) with the Newman–Keulspost test was applied. Statistical analysis was carried out using GraphPad Prism version 6.0 (San Diego, CA, USA). Differences were considered significant at *P *< 0.05.

## Results

### PI3K/AKT/mTOR pathway was activated in AAA clinical samples

**Fig. 1:**
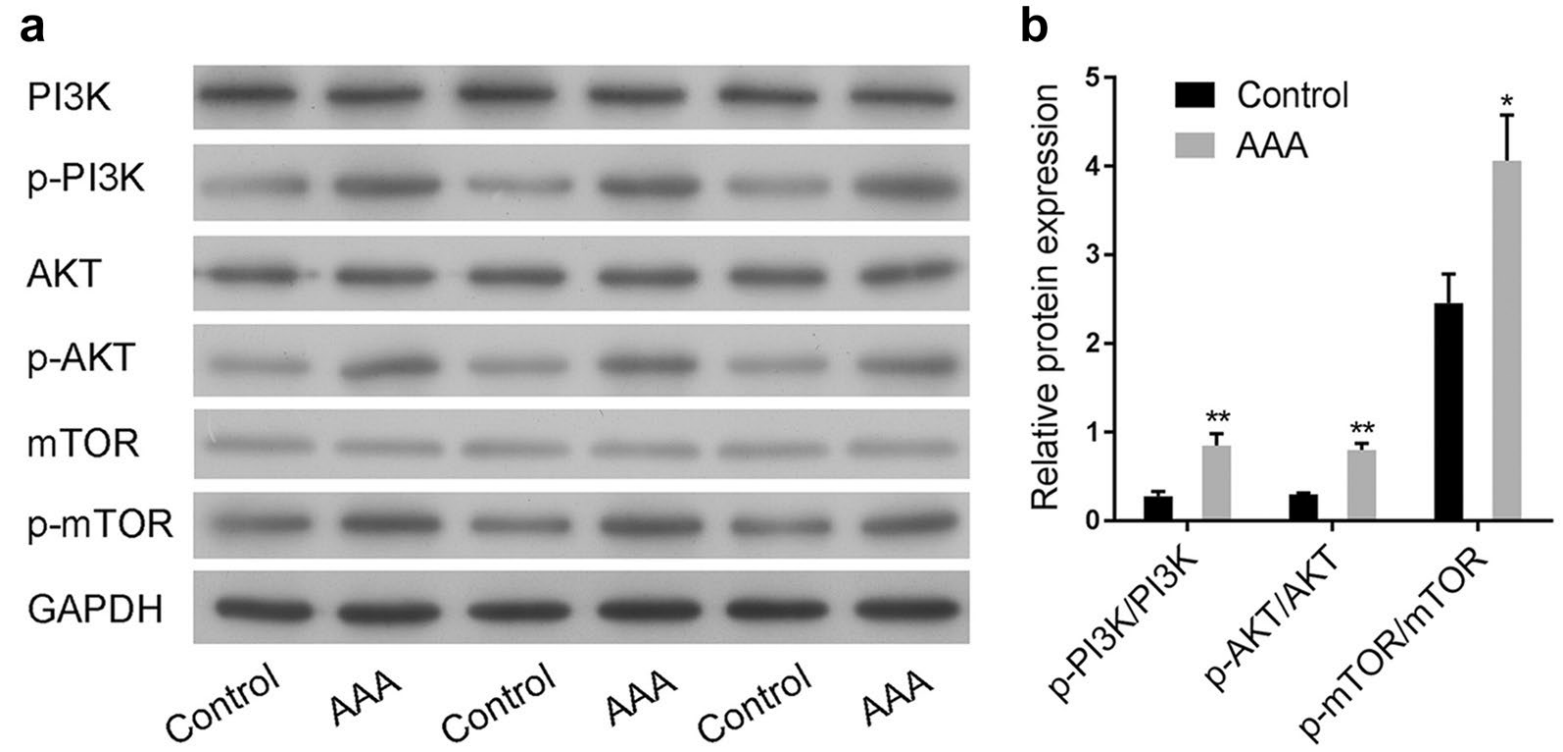
PI3K/AKT/mTOR pathway was activated in AAA clinical samples. **a** The changes of PI3K/AKT/mTOR pathway in the aneurysmal wall tissue of abdominal aortic aneurysm (AAA) or the normal aortic tissue (control) were detected by Western blot. **b** Semi-quantitative analysis of PI3K/AKT/mTOR pathway relative expression was performed

The activation of the PI3K/AKT/mTOR signaling pathway in aneurysmal wall tissues of AAA or the normal aortic tissues were explored using Western blot. Results showed that the phosphorylation levels of PI3K, AKT and mTOR in AAA were much higher than that in normal aortic tissues (Fig. 1a, b). These results suggested that PI3K/AKT/mTOR signaling pathway might be involved in the development of AAA.

### Metformin could attenuate the formation of Ang II-induced AAA in ApoE^−/−^ mice

**Fig. 2:**
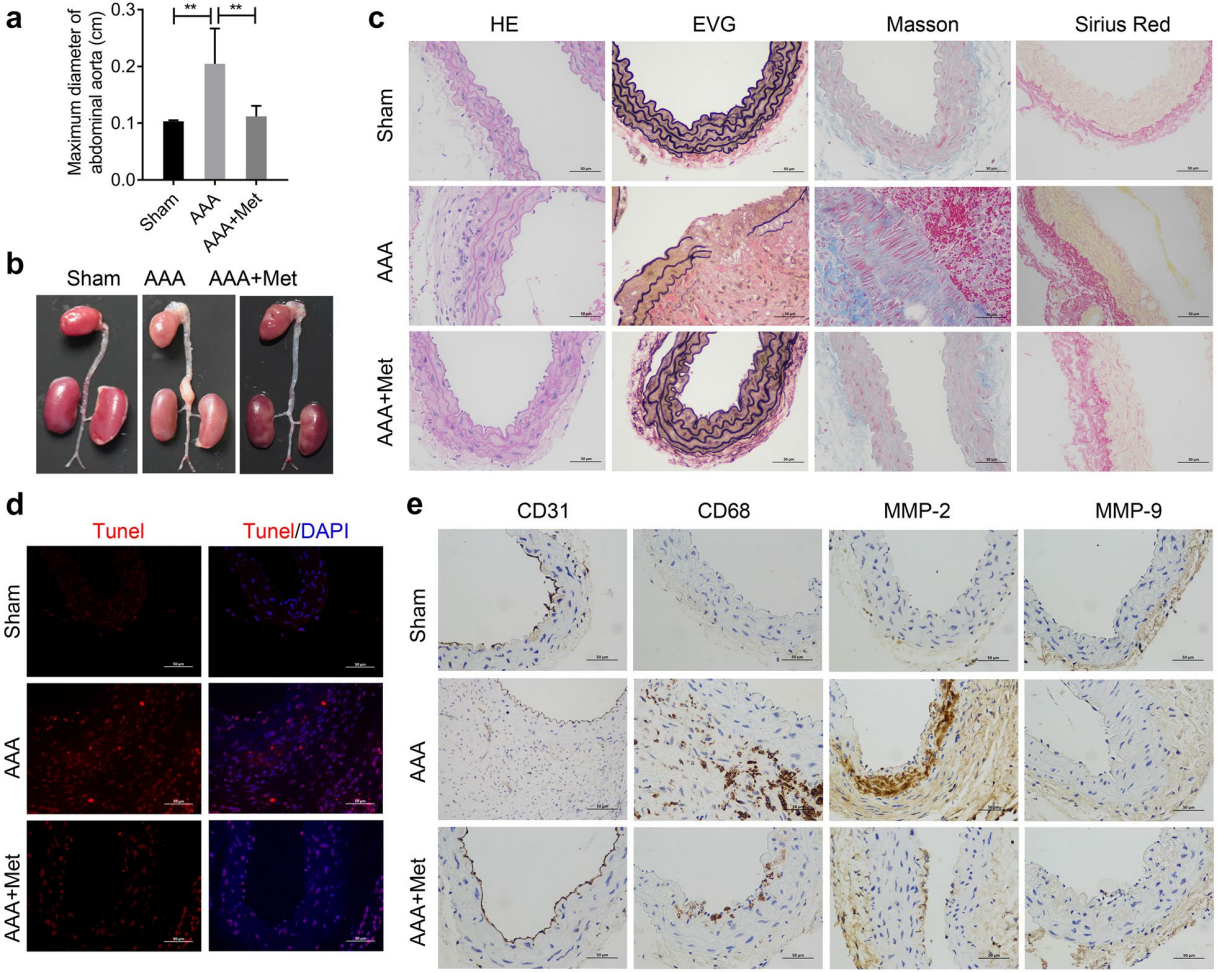
Metformin could attenuate the formation of Ang II-induced AAA in *ApoE*^−*/*−^mice. **a** Ultrasound was used to detect the maximum diameter of abdominal aorta in mice. **b** The abdominal aorta of mice in each group were removed surgically after 28 days of infusion and photographed. **c** The morphological changes of abdominal aorta in each group were observed using HE staining, EVG staining, Masson staining and Sirius Red Staining. **d** Tunel staining was used to detect the aortic tissue apoptosis. **e** Immunohistochemistry staining was used to observe the expression of CD31, CD68, MMP-2, and MMP-9 in aortic tissue

C57BL/6 mice at 10 weeks of age were divided into three groups: a) Sham group (N = 10, infused with normal saline); b) AAA group (N = 10, infused with Ang-II), and c) AAA and metformin group (N = 10, infused with Ang-II and feed water containing metformin). Ultrasound measurements of abdominal aorta diameter were performed on days 0 and 28 to determine the effect of model building. Ultrasound measurement results showed that Ang II perfusion for 4 weeks could significantly increase the maximum diameter of abdominal aorta in mice, while metformin could reverse this effect (Fig. 2a). The abdominal aortas of mice in each group were removed surgically on the 28th day. As shown in Fig. 2b, infusion with Ang-II promoted AAA formation in mice, while metformin reversed this effect (Fig. 2b). The tumorigenic rate was analyzed statistically after the experiment. The incidence of AAA in the Sham group, AAA group, AAA and metformin group, was 0%, 78% and 25%, respectively. All these data indicated that the construction of Ang-II-induced AAA mice model was successful. The morphological changes in abdominal aorta in each group were observed using HE staining, EVG staining, Masson staining and Sirius Red Staining, respectively (Fig. 2c). HE staining demonstrated that the AAA group exhibited a thicker vessel wall and more inflammatory cells infiltration in the media and adventitia of arteries when compared to the Sham group. However, these changes were attenuated by treatment with metformin. Elastin breakage of the arterial wall is an important step in the development of AAA [20]. EVG staining revealed the wavy structure of elastic lamellae in the sham group, indicating disruption and degradation in the AAA group. Treatment with metformin could effectively inhibit this breakage and preserve the elastin structure of the aorta. Masson staining further showed that the collagen which was dyed blue, was less abundant in the AAA group while muscular fiber, dyed red, was largely present in AAA group, indicating the presence of muscular fiber and loss of collagen. However, these changes were reversed after treating with metformin. Sirius Red staining also showed that collagen fiber was markedly increased in the AAA group compared to the Sham group. However, these changes were attenuated after treated with metformin. Tunel staining showed that there was more aortic cell apoptosis in the AAA group than in the Sham group, while metformin treatment could significantly attenuate Ang-II induced-apoptosis (Fig. 2d). Moreover, immunohistochemistry staining results indicated that the expression of CD31, CD68, and MMP-2 were upregulated in the AAA group when compared with the sham group, while these effects were partially reversed by treatment with metformin (Fig. 2e). The expression of MMP-9 in aorta tissues did not significantly changed (Fig. 2e). Overall, these results indicated that metformin could attenuate Ang-II-induced AAA formation.

### Metformin attenuates Ang-II-induced phosphorylation of PI3K/AKT/mTOR, and autophagy-related proteins expression in ApoE^−/−^ mice

**Fig. 3:**
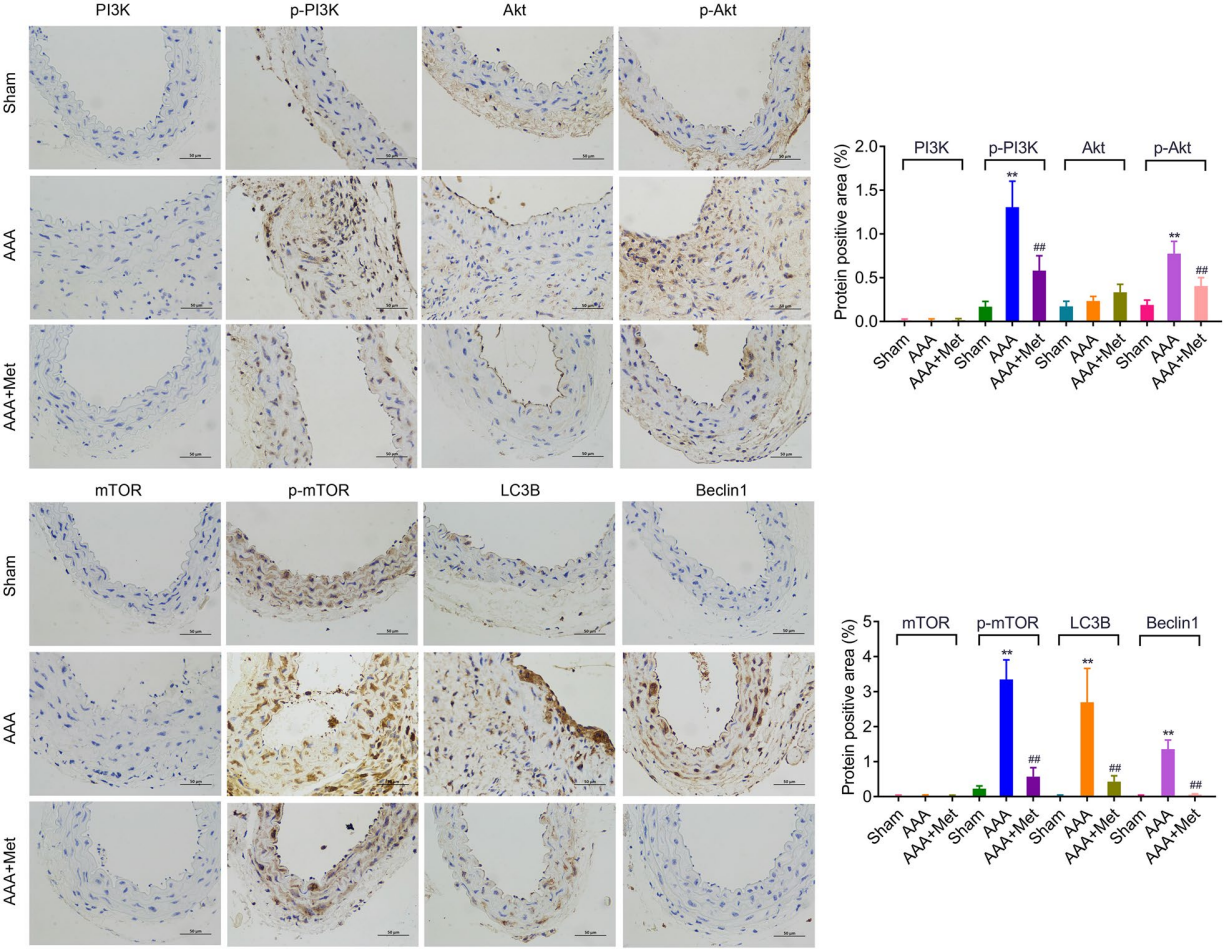
Metformin attenuates Ang-II-induced activation of PI3K/AKT/mTOR pathway and the expression of LC3B and Beclin1 in *ApoE*^−*/*−^mice. Immunohistochemistry staining was used to observe the expression of PI3K, AKT, mTOR, LC3B and Beclin1, and the phosphorylation levels of PI3K, AKT and mTOR in aortic tissue

**Fig. 4:**
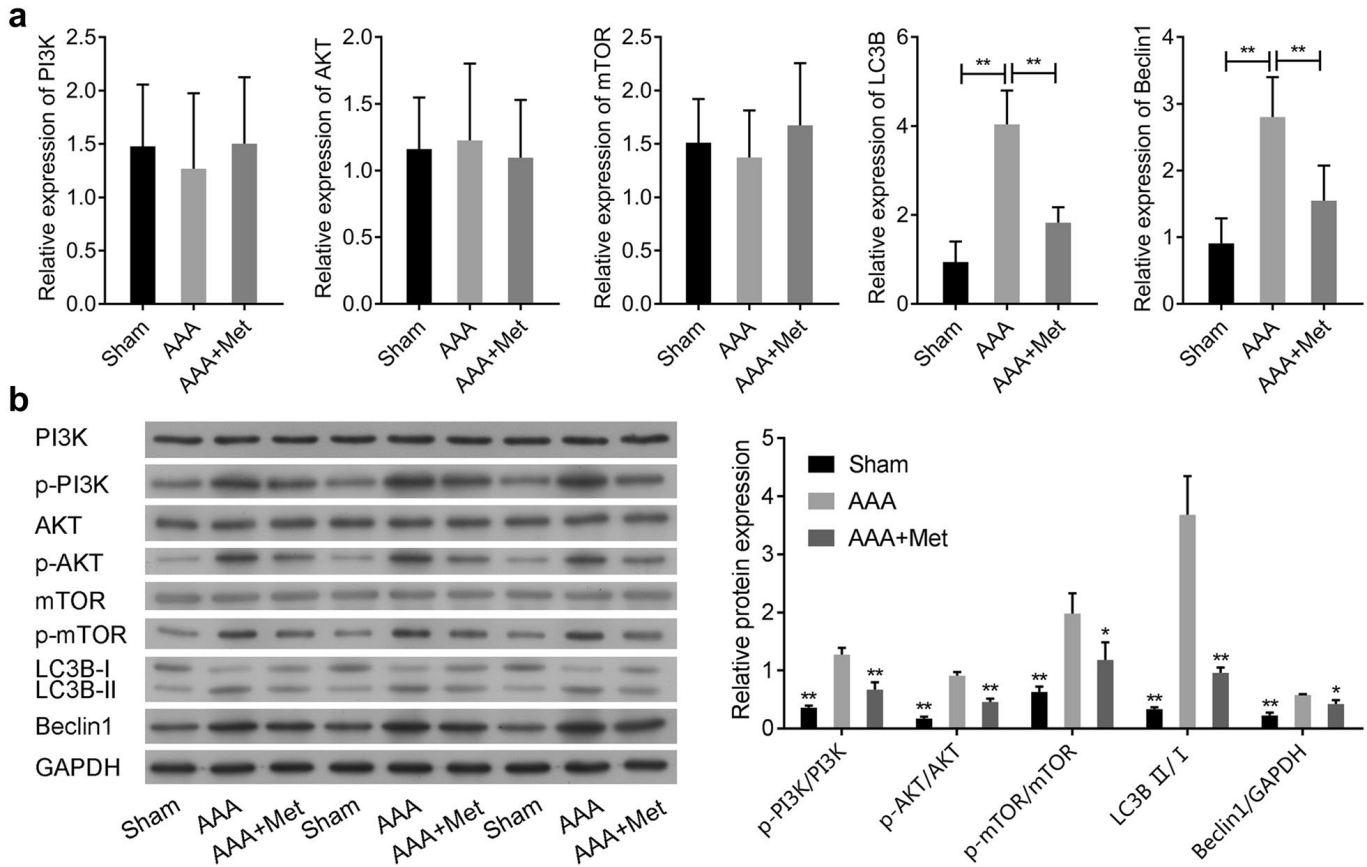
Metformin attenuates Ang-II-induced activation of PI3K/AKT/mTOR/autophagy pathway in *ApoE*^−*/*−^mice. **a** The mRNA levels of PI3K, AKT, mTOR, LC3B and Beclin1 in the aneurysmal wall tissue of abdominal aortic aneurysm (AAA) or the normal aortic tissue (Sham) were analyzed by qRT-PCR. **b** The protein levels of PI3K, AKT, mTOR, LC3B and Beclin1, and the phosphorylation levels of PI3K, AKT and mTOR were analyzed by Western blot

The aorta tissues in each group were isolated from ApoE^−/−^ mice after infusion with Ang-II for 28 days. Immunohistochemistry staining results indicated that the protein expression of LC3B and Beclin1 and phosphorylation levels of PI3K, AKT and mTOR were significantly upregulated in the AAA group compared with the Sham group (Fig. 3), while the expression of PI3K, AKT and mTOR in aorta tissues had no significantly change (Fig. 3). RNA analysis showed that Ang-II could significantly up-regulate LC3B and Beclin1 expression, but had no effect on the expression of PI3K, AKT and mTOR when compared to the Sham group (Fig. 4a). However, metformin treatment could dramatically reduce the expression of LC3B and Beclin1 induced by Ang-II (Fig. 4a). Moreover, Western blot analysis further confirmed that Ang-II up-regulated LC3B and Beclin1 expression, while these effects were partially reversed by treatment with metformin (Fig. 4b). The phosphorylation levels of PI3K, AKT and mTOR were significantly upregulated in AAA group when compared with the Sham group (Fig. 4b). Undoubtedly, the metformin treatment could markedly suppress this effect (Fig. 4b). These data indicated that PI3K/AKT/mTOR/autophagy pathway might be involved in the development of AAA.

### Metformin attenuates Ang-II-induced proliferation, apoptosis and autophagy in VSMCs

**Fig. 5:**
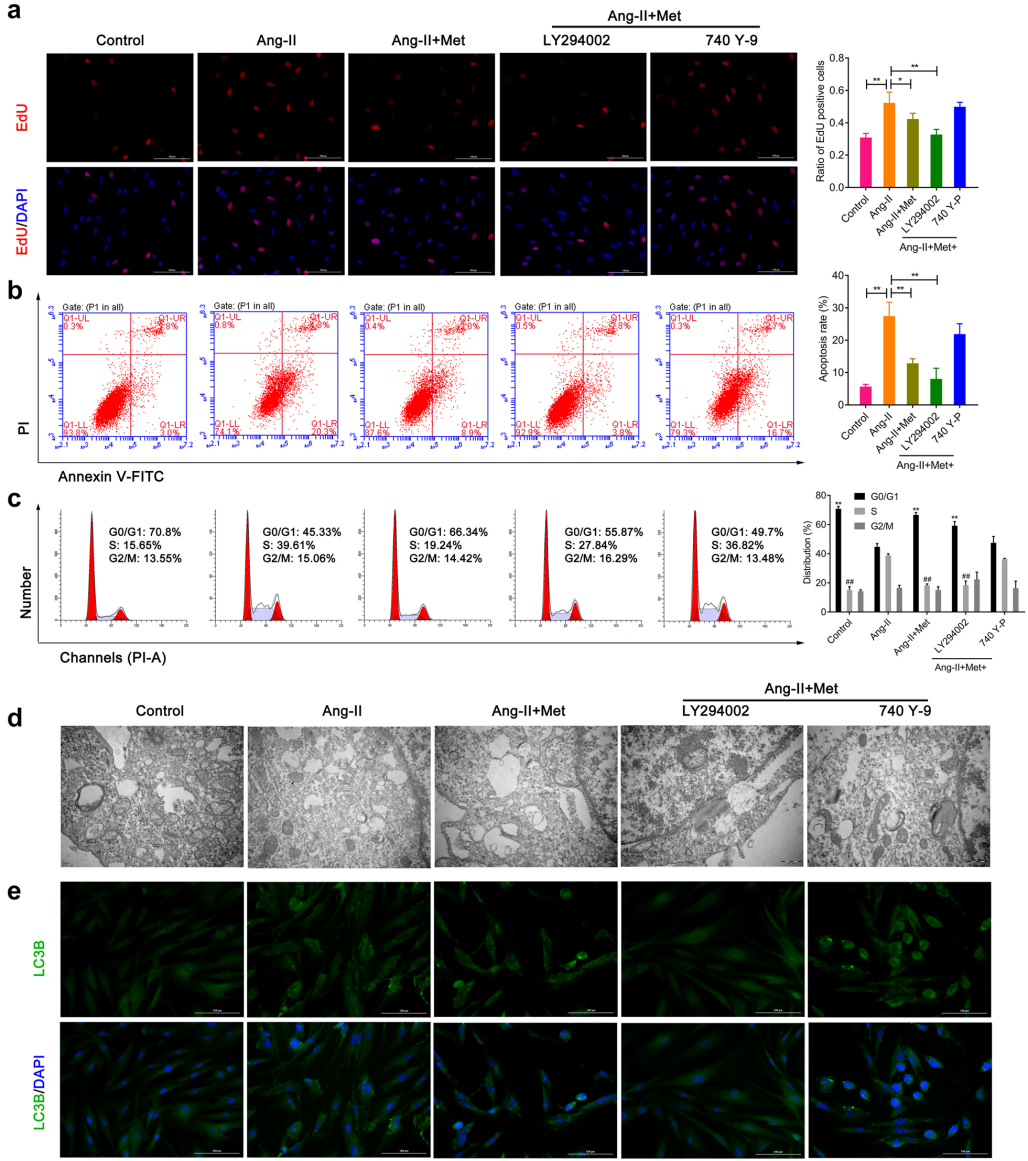
Metformin attenuates Ang-II-induced proliferation, apoptosis and autophagy in VSMCs. **a** EdU staining was used to detect the proliferation of VSMCs in five groups. **b**, **c** Flow cytometry was used to analyze the apoptosis and cell cycle of VSMCs. **d** Transmission electron micrographs demonstrate autophagosomes inside the cytoplasm of the VSMCs. **e** The expression of LC3B was detected by immunofluorescence

Before the cell experiments began, VSMCs were identified by immunofluorescence. As the data showed that α-SMA was highly expressed in VSMCs (Additional file 1: Figure S1A). Then VSMCs were divided into five groups and treated according to the method described above. EdU staining data showed Ang-II-induced proliferation markedly attenuated by metformin (Fig. 5a). PI3K inhibitors (LY294002) could effectively suppress the proliferation when compared with the Ang-II group, while these effects were largely reversed by PI3K activators (740 Y-P) treatment (Fig. 5a). Similar results were obtained in the apoptosis of VSMCs which was detected by flow cytometry (Fig. 5b). Moreover, Similar results were obtained in the expression of Bax in VSMCs detected by western blot, while the expression of Bcl-2 in VSMCs showed an opposite trend (Additional file 1: Figure S1B). Cell cycle results further demonstrated that Ang-II could increase S-phase cell ratio, while metformin could markedly attenuate Ang-II -induced S-phase cell ratio in VSMCs (Fig. 5c). However, there was an opposite trend in regulation of G0/G1 phase (Fig. 5c). All of these data indicated that metformin inhibited Ang-II-induced cell proliferation through G0/G1 phase cell cycle arrest in VSMCs. Transmission electron micrographs further illustrated a greater increase in autophagosomes inside the cytoplasm of the VSMCs when compared with the control group treated with Ang-II. However, metformin treatment could attenuate the effect of Ang-II on autophagy. In addition, LY294002 partially attenuated autophagosome presence, while 740 Y-P largely increased the autophagosome presence inside the cytoplasm (Fig. 5d). The results of immunofluorescence detection of LC3B were similar to those obtained by transmission electron micrographs (Fig. 5e).

### Metformin inhibits Ang-II-induced migration of VSMCs

**Fig. 6:**
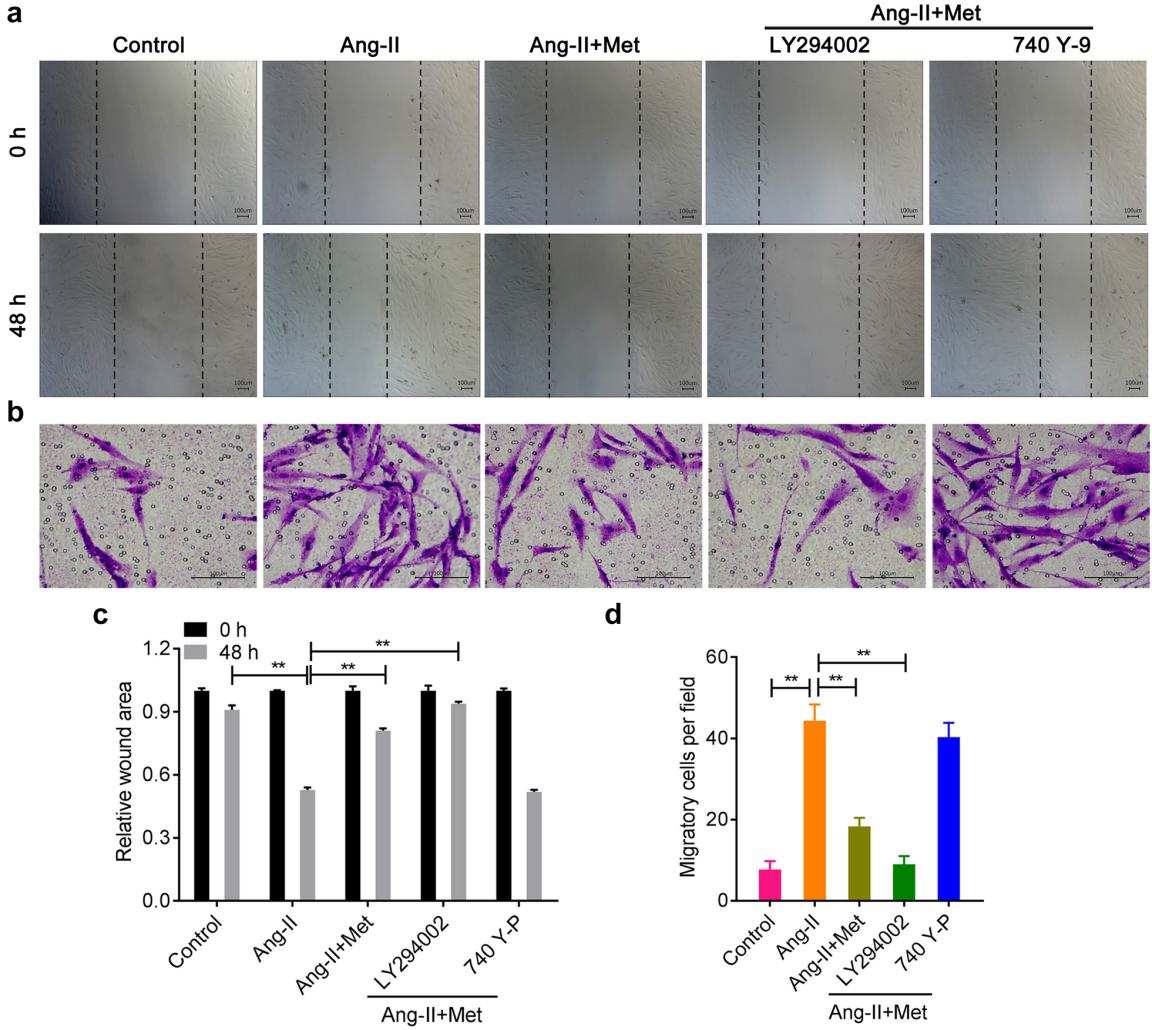
Metformin suppressed Ang-II-induced migration of VSMCs. The wound healing assay (**a**, **c**) and transwell chamber migration assay (**b**, **d**) were used to analyze the migration of VSMCs

In Fig. 6, both the wound healing assay and transwell assay showed that, Ang-II promoted the migration of VSMCs, while metformin effectively inhibited this effect (Fig. 6a–d). LY294002 partially increased the inhibitory effect of metformin on Ang II-induced migration in VSMCs, while 740 Y-P produced the opposite trend (Fig. 6a–d).

### Metformin regulates the function of VSMCs by regulating the PI3K/AKT/mTOR/autophagy associated proteins

**Fig. 7:**
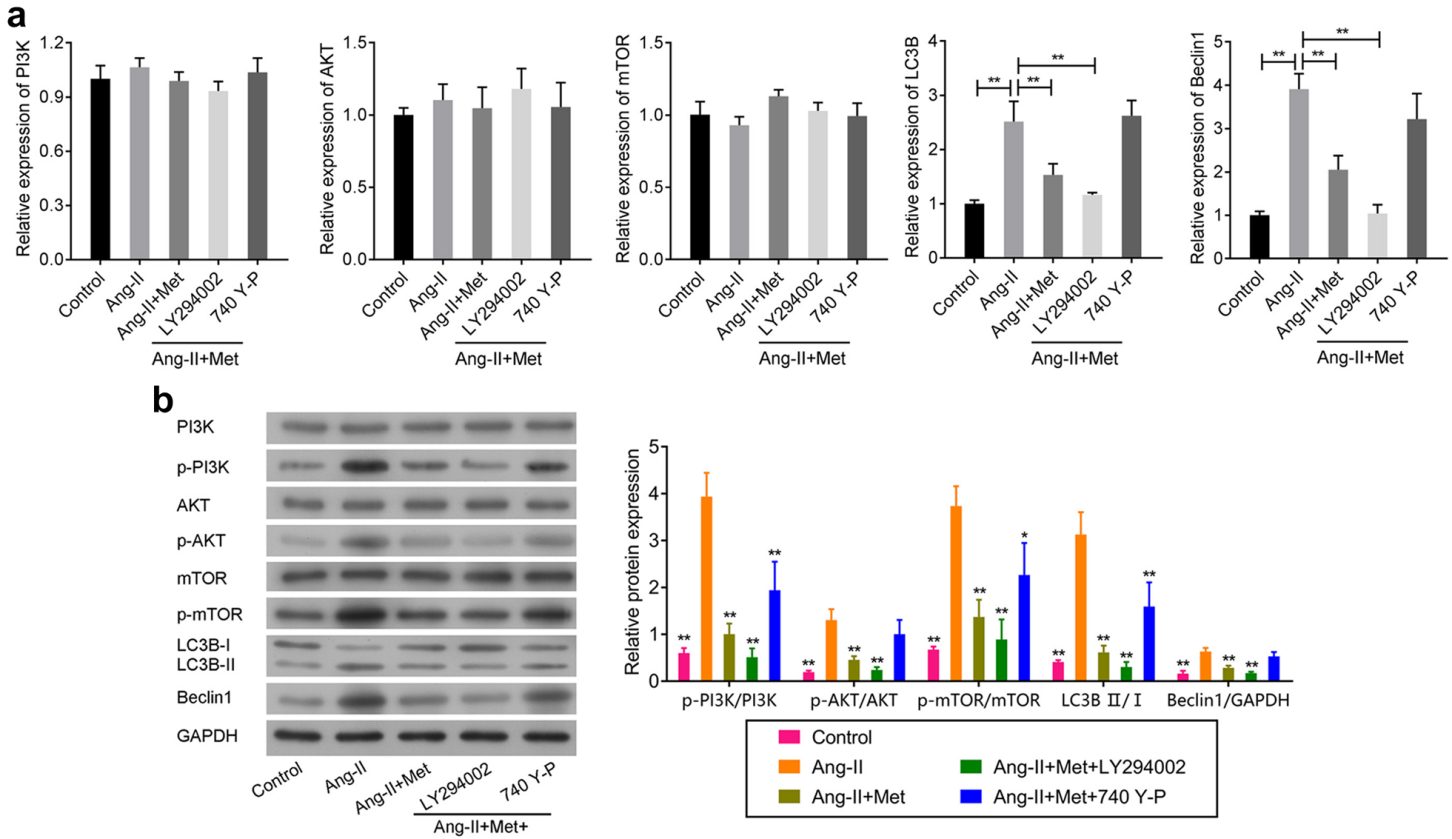
Metformin regulated the function of VSMCs through mediating the PI3K/AKT/mTOR/autophagy pathway. The effect of metformin on the PI3K/AKT/mTOR pathway and autophagy associated proteins in Ang-II-induced VSMCs were explored by qRT-PCR (**a**) and Western blot (**b**). Rat abdominal aortic SMCs were firstly treated with the PI3K inhibitors (20 mM) and PI3K agonists (25 mM) for 24 h in these experiments, respectively

Using qRT-PCR and Western blot, we further confirmed whether metformin influenced the pathogenesis of AAA via regulating the PI3K/AKT/mTOR pathway and autophagy related proteins. As shown in Fig. 7a, there were no significant changes in PI3K, AKT and mTOR mRNA expression. But for LC3B and Beclin1 mRNA, the mRNA expression levels induced by Ang-II were significantly suppressed by metformin. This effect was markedly enhanced by PI3K inhibitors and largely reversed by PI3K agonists (Fig. 7a). Interestingly, we obtained similar results for LC3B and Beclin1 protein expression (Fig. 7b). In addition, we also observed that phosphorylation of PI3K, AKT and mTOR was significantly activated by Ang-II (Fig. 7b). However, metformin could substantially suppress these phenomena. Moreover, the PI3K inhibitors strengthened this effect while PI3K agonists showed the opposite trend. All of these data indicated that metformin regulated the function of VSMCs by affecting the PI3K/AKT/mTOR pathway and autophagy associated proteins.

## Discussion

AAA which is very common in elderly patients is associated with serious complications and greatly increases morbidity risk. The current standard of treatment for AAA mainly includes surgical and interventional treatment. However, these two treatment methods have certain limitations. Surgical treatment produces substantial physiological trauma. In elderly patients receiving interventional treatment, the diagnosis of AAA is often late; affected blood vessels are not limited to the aortic vessels, which limits the applicability of interventional therapy in elderly patients [21]. Therefore, AAA pathogenesis, prevention, and treatment strategies have always been the focus of cardiovascular disease research.

Many studies have demonstrated that the degradation of extracellular matrix in aorta and the depletion of medial smooth muscle cell often lead to the occurrence of AAA [3, 22, 23]. As the main component cell of abdominal aorta media, smooth muscle cells (SMCs) directly or indirectly secrete elastin, collagen and other matrix proteins, which play a very important role in the construction and repair of arterial elastic lamella. Therefore, we chose VSMCs as the main cell to further explore how metformin represses the development of AAA.

**Fig. 8:**
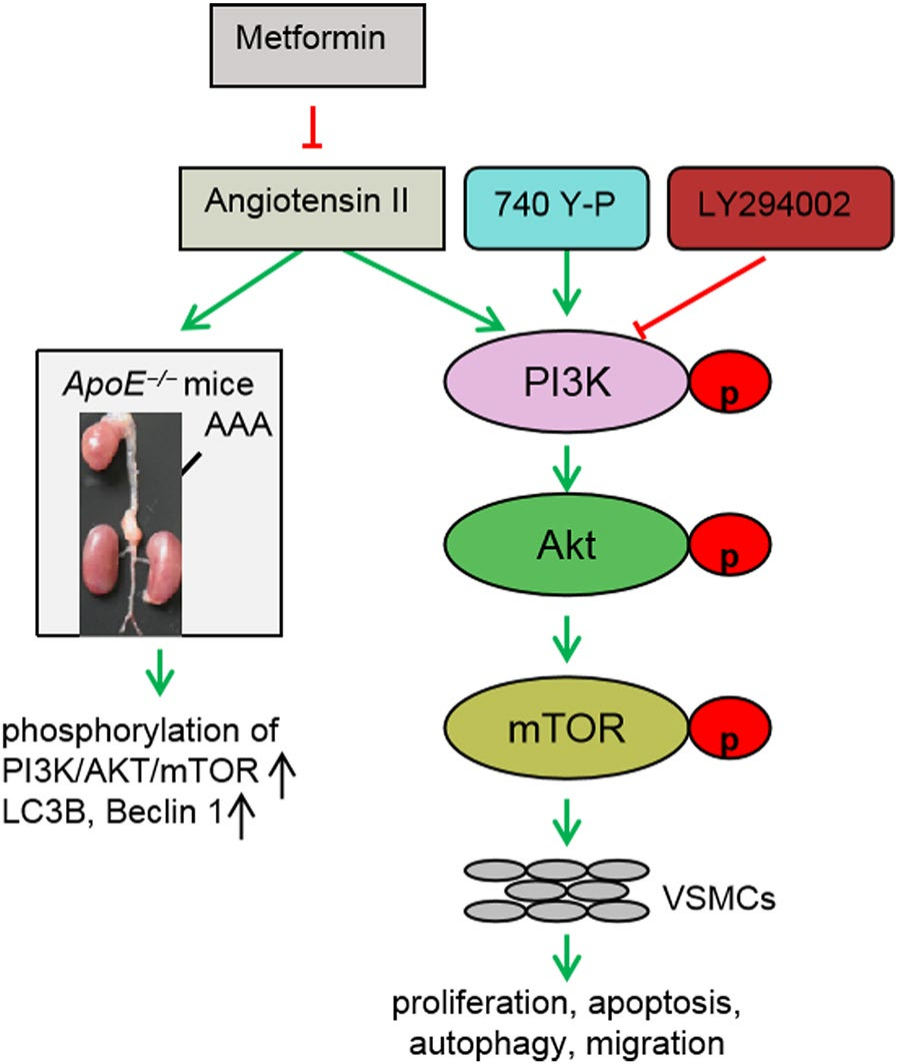
The schematic diagram of the mechanism by metformin represses the Ang-II-induced pathophysiology of AAA by regulation the PI3K/AKT/mTOR/autophagy pathway

Metformin is the most widely prescribed drug for the treatment of hyperglycemia in patients with type 2 diabetes [24]. As a new kind of old medicine, it has been widely used in various tumors, including lung cancer, breast cancer, colon cancer and thyroid cancer [25–28]. Metformin inhibits mTOR phosphorylation by activating AMPK in the AMPK/mTOR signaling pathway, thereby leading to the arrest of tumor cell cycle and the inhibition of cell growth and proliferation, which finally results in cell apoptosis [29]. Here, we demonstrated that mTOR phosphorylation was substantially up-regulated in both AAA patient tissues and AAA rat model. However, treatment with metformin significantly reversed this phenomenon in the AAA rat model. PI3K/AKT signaling pathway plays an important role in cell survival, differentiation, proliferation and apoptosis. PI3K-AKT pathway dysfunction is present in many tumors, such as lung cancer, liver cancer, breast cancer, ovarian cancer, prostate cancer and so on [30–34]. In addition, researchers have demonstrated that this pathway is related to many non-tumor diseases including liver fibrosis, Alzheimer’s disease, diabetes and cardiovascular disease [35–38]. Selective inhibition of the PI3K/AKT signaling pathway can promote autophagy of macrophages, reduce the infiltration of plaque macrophages, suppress the inflammatory response, and thereby stabilize atherosclerotic plaques [39]. Reports also demonstrated that PI3K/AKT is an important signaling pathway for growth factors or cytokines involved in VSMCs proliferation and migration [40]. Meanwhile, PI3K/Akt signaling could be differentially activated to regulate vascular remodeling and VSMCs proliferation and migration. Given that the main pathological features of AAA are the increase in inflammatory cell infiltration, the degradation of extracellular matrix and the apoptosis of VSMCs, we also selected this pathway to explore the influence on AAA progression. We observed that the phosphorylation of both PI3K and AKT was significantly activated in AAA, but that these phenomena were largely suppressed by metformin treatment. In addition, we also found that the proliferation, migration and autophagy induced by Ang-II in rat abdominal aortic smooth muscle cells were obviously suppressed by metformin treatment. The PI3K inhibitors LY294002 further enhanced these effects, but PI3K agonists largely reversed these phenomena. These results revealed that metformin played a regulatory role in the biological behavior of Ang-II-stimulated rat abdominal aortic smooth muscle cells through the PI3K/AKT/mTOR pathway (Fig. 8). However, we found that there was no difference in the progression of PI3K, AKT and mTOR between AAA tissues and normal tissues. We also confirmed that the mRNA levels of PI3K, AKT and mTOR VSMCs showed no significant changes whenever treated with metformin and/or Ang-II. All of these results indicated that metformin regulated the activation of PI3K/AKT/mTOR pathway through phosphorylation level during the pathological process of AAA, rather than through mRNA level and total protein level. These conclusions need further verification.

The apoptosis of VSMCs leads to the occurrence of AAA [41]. It is particularly important to study the apoptotic mechanism of VSMCs for the prevention and treatment of AAA. Inhibition of VSMCs apoptosis can reduce inflammatory response, cytokine activation and the abnormal proliferation of VSMCs in injury region [42]. Therefore, reducing the apoptosis rate of VSMCs is the key to inhibit the formation and progression of AAA. Here, we also demonstrated that the apoptosis rate, which was substantially induced by Ang-II, was correspondingly decreased by metformin stimulation. Interestingly, reports have demonstrated that NOX enzymes, which up-regulate the ROS level to initiate and maintain tumorigenesis, are involved in apoptosis-induced proliferation (AiP) through regulating the PI3K/AKT/mTOR pathway [43]. The possible role of metformin in regulating AiP by mediating the mitochondrial ROS requires further exploration. Interestingly, many reports have proved that Angiotensin II could promote the proliferation, hypertrophy and apoptosis of VSMC [44]. Here, we also proved that Ang-II not only accelerated the proliferation of VSMCs but also promoted the apoptosis of VSMCs. This phenomenon might be due to the compensatory proliferation of VSMCs caused by Ang-II treatment, which means the apoptotic VSMCs induced by Ang-II releases certain signals and promotes the proliferation of surrounding cells.

Reports have confirmed that the matrix metalloproteinases produced by macrophages and SMCs can cause elastin and collagen degradation, which is closely related to the occurrence of AAA [45, 46]. MMP-2 and MMP-9 are the main proteases that degrade elastic fibers in AAA, resulting in the formation and development of AAA [45, 46]. In this study, we found that MMP-2 was not expressed in normal aortic tissues, but highly expressed in AAA tissues. It is possible that the stimulation of SMC by infiltrating lymphocytes and macrophages in the wall of AAA leads to the increase of MMP-2 expression. However, further study is needed to evaluate MMP-2 expression of SMCs when SMCs of aortic wall, and infiltrating macrophages and lymphocytes in AAA tissues are cocultured. We found no significant difference in MMP-9 between AAA and normal tissues, suggesting that MMP-9 might not participate in pathological processes of this AAA model; this also requires further confirmation.

As a transmembrane glycoprotein, CD31 is also a self-interacting molecule expressed on both cells of the innermost layer of vascular wall and on leukocytes [47, 48]. In addition, the signals suppressed by CD31 might play a key role in regulating vascular immune-mediated pathologies [48]. Here, we found that CD31 expression was highly expressed in AAA clinical tissues and the AAA model, meaning that CD31 might participate in vascular immune-mediated pathologies during the occurrence of AAA. The inflammation and matrix degradation of AAA vascular wall are the key factors causing AAA [9, 10]. Therefore, we further detected the changes of macrophage marker CD68 and confirmed that CD68 expression was significantly up-regulated in AAA, and this was largely suppressed by metformin treatment. These results further indicated that metformin regulated AAA by depressing the inflammatory response. Autophagy, as another form of programmed cell death, is highly conserved in evolution. Autophagy is involved in the degradation of most proteins that have long half-lives. Recently, more and more studies have shown that autophagy or apoptosis is induced in different cells under the same inducing factors. Terman et al. proved that myocardial autophagy plays an important role in the cardiomyopathy [49]. Studies also demonstrated that vascular autophagy is present in atherosclerosis and is regulated by tumor necrosis factor-alpha and insulin-like growth factor-1 [50]. LC3 and Beclin1 are common biomarkers of autophagy, which are used to evaluate the level of autophagy. LC3, as a universal marker of autophagy vesicles, is located on the surface of pre-autophagy and autophagy vesicles [51]. LC3 modification is essential for the formation of autophagic vesicles. For Beclin1, the main mechanism in autophagy mainly involves the recruitment of proteins to form a complex with PI3K, thereby promoting the formation of autophagosome membrane and guiding other Atg proteins to localize in the autophagosome membrane [52, 53]. Here, we found that the proportion of autophagy protein LC3B and Beclin1 in VSMCs increased following the Ang-II treatment, but this effect was largely reversed by metformin and enhanced under the co-stimulation of PI3K inhibitors, indicating that VSMCs autophagy is present in AAA progression and that metformin might reduce this phenomenon by mediating the PI3K/AKT pathway. However, we still needed using the inhibitors and inducers of autophagy to further confirm the effects of metformin on the autophagy during the AAA progression and also further explored the related mechanism.

More and more evidences have proved chronic inflammatory response plays an important role in the development of AAA. Except releasing multiple MMPs which promote the degradation of tubular structural proteins (collagen, laminin, elastin), activated inflammatory cells could also secrete a variety of inflammatory factors to accelerate the migration and activation of inflammatory cells, which causing the continued and amplified local inflammatory response [54, 55]. Studies have demonstrated that local and systemic inflammatory cytokines and chemokines including TNF-α, IFN-γ, IL-1β, IL-6, IL-17 are significantly up-regulated [56]. Besides, inflammatory cytokines also act on endothelial cells and smooth muscle cells through autocrine and paracrine modes, which causing phenotypic changes and inducing immune response to promote apoptosis of smooth muscle cells [57, 58]. Therefore, we also detected the influences of metformin on the inflammation in another research. In conclusion, our study revealed that metformin might repress the occurrence and development of AAA at least partially through inhibition of activation of the PI3K/AKT/mTOR pathway.

## Supplementary information

**Additional file 1: Figure S1.** The expression of α-SMA, Bcl-2 and Bax in VSMCs. (A) Immunofluorescence staining was used to detect the expression of α-SMA in VSMCs. (B) The expression of Bax and Bcl-2 in VSMCs were detected by western blot analysis.

## Data Availability

The datasets generated/analyzed during the current study are available.

## Abbreviations

AAA: abdominal aortic aneurysm
Ang-II: angiotensin II
CT: computerized tomography
Edu: 5-ethynyl-2′-deoxyuridine
EVG: elastica van Gieson
HE: hematoxylin and eosin
IHC: immunohistochemistry
Met: metformin
MRI: magnetic resonance imaging
qRT-PCR: quantitative real-time PCR
TEM: transmission electron microscope
TUNEL: TdT-mediated dUTP nick end labeling
VSMCs: vascular smooth muscle cells
PI3K: phosphoinositide 3-kinase
mTOR: mammalian/mechanistic target of rapamycin
MMP-3: matrix metalloproteinase-3
MMP-9: matrix metalloproteinase-9
