# Supplementary figures and images for: Metformin represses the pathophysiology of AAA by suppressing the activation of PI3K/AKT/mTOR/autophagy pathway in ApoE−/− mice
Source: Cell Biosci. 2019 Aug 27;9:68. doi: 10.1186/s13578-019-0332-9 (PMC6712653; doi:10.1186/s13578-019-0332-9)

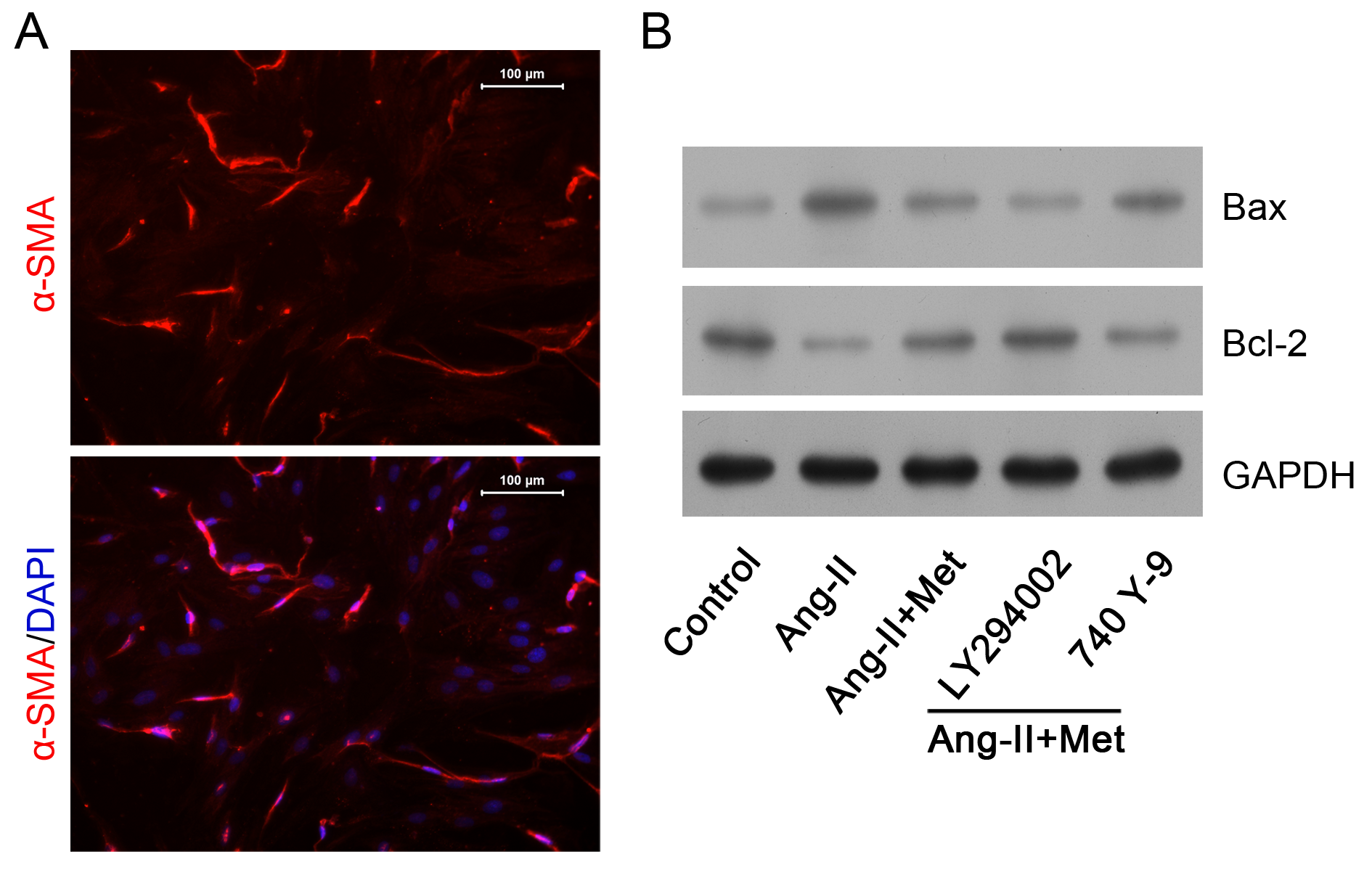

Supplement: Supplementary file 1 — Additional file 1: Figure S1. The expression of α-SMA, Bcl-2 and Bax in VSMCs. (A) Immunofluorescence staining was used to detect the expression of α-SMA in VSMCs. (B) The expression of Bax and Bcl-2 in VSMCs were detected by western blot analysis. [file 13578_2019_332_MOESM1_ESM.tif]
